# New Tumor-Inhibiting Metal Complexes. Chemistry and Antitumor Properties

**DOI:** 10.1155/MBD.1994.145

**Published:** 1994

**Authors:** B. K. Keppler, M. Hartmann

**Affiliations:** Anorganisch-Chemisches Institut Universität Heidelberg Im Neuenheimer Feld Heidelberg D - 69120 Germany


### NEW TUMOR-INHIBITING METAL COMPLEXES. CHEMISTRY AND ANTITUMOR PROPERTIES

B.K. Keppler* and M. Hartmann

Anorganisch-Chemisches Institut, Universität Heidelberg, Im Neuenheimer Feld
D - 69120 Heidelberg, Germany

Metals such as platinum, gold, ruthenium, titanium and tin can be a source of biologically active derivatives and, occasionally, become tools in the chemotherapy against different kinds of human tumors. The preclinical evaluation includes not only antitumor activity tests but also the investigation of the particular chemistry, the molecular targets within biological materials and the effect on the survival of a cell within its particular environment.

These metals may form coordination compounds by binding ligands **firmly** or **loosely**, depending on the chemical nature of both the metal ion and the ligands. A **loosely** coordinated chloride ion or water molecule will exchange for a more nucleophilic biomolecule, commonly a nucleobase or a sulfur-containing amino acid side chain, and thereby infer a biological lesion. A metal ion complex may require an exchange of one or more ligands for water in order to become activated. All the **firmly** bound, conserved, ligands influence both target selection and reactivity of the metal ion. Following the important thesis of Collier and Krauss[1] from the 30s ("The effect of a heavy metal on experimental murine cancer is not only due to the metal alone, but also to the structure of the compounds and the type of compound"), it should be emphasized that the efficacy of a metal complex against cancer is primarily related to the kind of its set of ligands and to the resulting substitution stability of the different bounded ligands.

Under this aspect, two new tumor-inhibiting metal complexes will be discussed in this article, the ruthenium complex *trans*-Indazole-tetrachlorobis(indazole)ruthenate(III) and the titanium complex Budotitane (INN), *cis*-diethoxybis(1-phenylbutane-1,3-dionato)titanium(IV) (Fig. 1).

Both compounds show the best activity in experimental tumor models among a huge amount of corresponding derivatives that we have synthesized and tested before. Budotitane is now under clinical studies in patients, and the ruthenium indazole complex is at an highly advanced preclinical stage of development and will enter clinical studies soon. Another important ruthenium complex

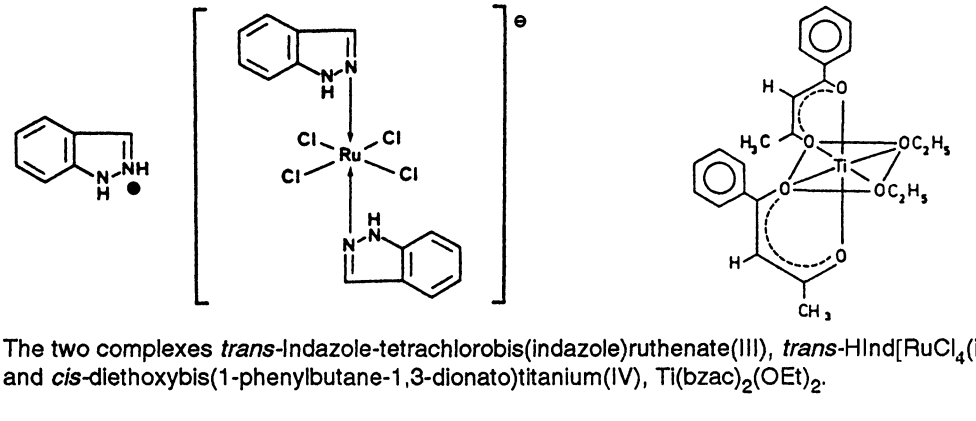

Fig. 1: The two complexes *trans*-Indazole-tetrachlorobis(indazole)ruthenate(III), *trans*-HInd[$RuCl_4(ind)_2$], and *cis*-diethoxybis(1-phenylbutane-1,3-dionato)titanium(IV), $Ti(bzac)_2(OEt)_2$.

[1]W.A. Collier, F. Krauss, Zeitschrift für Krebsforschung, 1931, *34*, 527

with two *trans*-standing imidazole ligands, *trans*-HIm[$RuCl_4(im)_2$], ICR, that shows nearly as excellent results as the indazole compound, will be considered as well. For the selection of most active complexes, a procedure has been developed in Heidelberg which is based on the combination of transplantable and autochthonous tumor models. After synthesis and chemical characterisation, the compounds are compared on the basis of activity in various transplantable tumor models. These models are chosen in such a way that most of the compounds from the new types of substances to be examined show medium activity in each relevant model, with the result that complexes with major or minor activity can clearly be distinguished. After optimising the chemical structure, compounds with outstanding activity are further investigated in autochthonous tumor models. We selected AMMN-induced colorectal tumors of the rat for our antitumor activity tests. This autochthonous model has a high predictivity for the clinical situation (Fig. 2). Colorectal tumors are responsible for the major share of total cancer mortality among humans today.

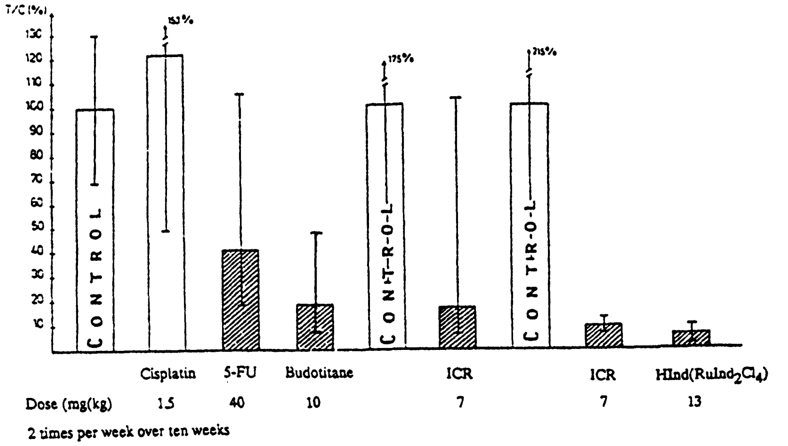

Fig. 2: Test results of Budotitane and HInd[$RuCl_4(Ind)_2$] against this model are shown, compared to cisplatin and 5-fluorouracil.

Cisplatin, *cis*-Diamminedichloroplatinum(II), turned out to be totally ineffective, as is the case in patients suffering from this kind of tumor. Budotitane and HInd[$RuCl_4(ind)_2$] show highly promising effects against this important type of tumor, similar or even better than fluorouracil, which is the only drug to exhibit activity against this tumor in human cancer patients.

In the case of cisplatin, which is clinically active mainly against testicular carcinomas and few other types of tumors, the kinetical stability of the complex is an outstanding criterion for its use as a therapeutical agent. A prevention of fast hydrolysis reactions leads our attention to other metals that show hydrolysis stability similar to that of platinum(II). Ruthenium(III) is well known for its stability in aqueous solution; for the tumor-inhibiting complexes like HInd[$RuCl_4(ind)_2$] and HIm [$RuCl_4(im)_2$], a slow exchange of their ligands can be predicted (Fig. 3).

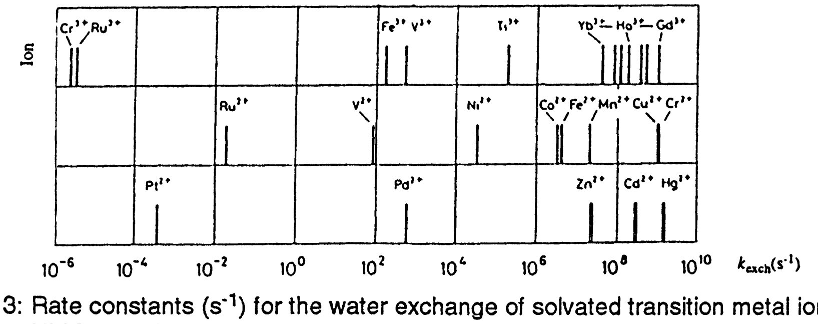

Fig. 3: Rate constants ($s^{-1}$) for the water exchange of solvated transition metal ions, measured directly by NMR or estimated from the rate constants for complex formation ( — NMR ═ complex formation). (Following Y. Ducommun and A.E. Merbach in Inorg. High Pressure Chem., Elsevier, 1986, *B 17*)

HPLC investigations of HInd[$RuCl_4(ind)_2$] in $H_2O$ showed indeed a slow decomposition (in physiological saline at 22°C) with a rate of hardly 1% per hour. The initial formation of an aqua complex could also be confirmed by the isolation of the complex aquatrichloro(1-methyl-indazole)ruthenium(III), [$Ru(H_2O)Cl_3(1MeInd)_2$]$^-$. The UV spectra of the aqua complex show a characteristic absorption at 360 nm, which misses in the spectra of the initial complex 1MeInd [$RuCl_4(1MeInd)_2$]. This absorption was the basis of the assignment of the aqua complexes formed during hydrolysis of HInd[$RuCl_4(ind)_2$]. The imidazole complex hydrolizes faster in physiological saline (22°C); after one hour nearly 3 % of the original complex has been substituted in solution. The respective imidazolium ion is the stronger base and leads to a less acidic reaction milieu, the concentration of nucleophiles (imidazole, chloride and $H_2O$) is raised. The result is the observed loss of stability of the imidazole complex in physiological saline (Fig. 4). A general decrease in stability can be observed when HInd[$RuCl_4(ind)_2$] is added to a physiological buffer solution (0.1 M NaCl, 0.004 M $NaH_2PO_4$, 0.025 M $NaHCO_3$). A blue-green precipitate is formed after 10 minutes (37°C). These investigations have been done in preparation of experiments that showed us the interaction of HInd [$RuCl_4(ind)_2$] and HIm[$RuCl_4(im)_2$] in human serum. The UV and HPLC studies of the reactions in phys. buffer showed us that the imidazole complex is much more stable than the corresponding indazole compound. This is not surprising in view of the stronger basicity of the imidazole ligands. Assoziative nucleophilic substitutions occur normally in the coordination sphere of ruthenium(III), a higher electron density at the metal hinders a nucleophilic attack.

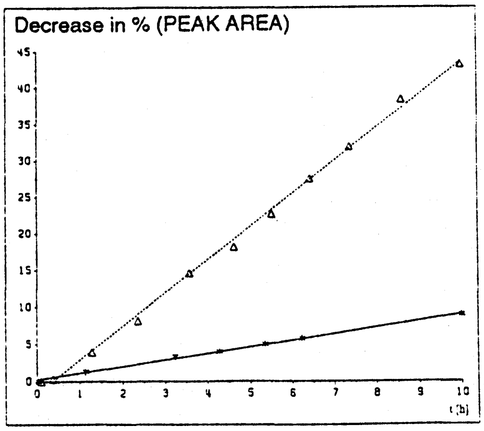

Fig. 4: HPLC investigations into the hydrolytic decomposition of HIm[$RuCl_4(im)_2$] (= Δ) and HInd[$RuCl_4(ind)_2$ ] (= •) in phys. saline at a temperature of 22°C.

To obtain an insight into the molecular modes of intravenously administered antitumor metal complexes, it is important to study the interactions of those with plasma proteins. For example, the differences in efficacy, activity and toxicity between cisplatin and carboplatin are discussed in relation to the measured differences in the reversibility of plasma binding of both compounds.

LPLC studies of HInd[$RuCl_4(Ind)_2$] showed that the major amount of the complex is bound to albumin in serum (about 80 %). This is not surprising when we consider the amount present in serum (approximately 42 weight per cent of the solutes) and the size of albumin. A small amount of HInd[$RuCl_4(Ind)_2$] is bound to transferrin, too. Ultrafiltration experiments have given evidence that no binding to low molecular species (< M.W. 30.000) occurs. HPLC studies of the reaction rate showed that binding to transferrin occurs within 5 minutes. After addition of citrate to the transferrin adduct, free ruthenium complex could be observed in the HPLC that demonstrates the reversible character of the binding. More details will be given in the abstract by *F. Kratz* in this book.

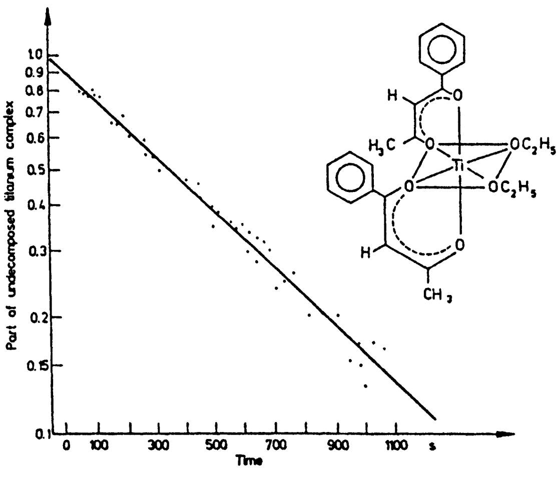

Fig. 5: Pseudo first order decay of $Ti(bzac)_2(OEt)_2$ during hydrolysis of 20 mg of complex in 400 µl of $CD_3OH$ + 20 µl of $D_2O$ at 22°C.

Budotitane, in contrast, belongs to the class of rapidly hydrolysing metal complexes. If a $10^{-3}$ M solution of $Ti(bzac)_2(OEt)_2$ is dissolved in absolute acetonitrile, the addition of 20 vol.% $H_2O$ leads to the decomposition of the complex within a few minutes. A corresponding experiment is shown in Fig. 5, it contains the measurement of the decomposition of budotitane by the NMR signal of the Ti-O-$CH_2$-$CH_3$ protons. The primary product of the hydrolysis of the two ethoxy groups of budotitane can be obtained in a pure form by reaction of the complex with water in the stoichiometric ratio of 1:2 in acetonitrile and subsequent evaporation of the solvent and recrystallisation from $CCl_4$ / petroleum ether. Its composition is $[Ti(bzac)_2O]_n$, which is probably produced by oligomerisation of the intermediate $Ti(bzac)_2(OH)_2$ that is formed on the reaction of budotitane with water. The molecular weight (between n = 2 and n = 3) could be confirmed by vapour pressure osmometric measurements. The much higher decomposition rate of budotitane has consequences for the galenic formulation of the complex. A drug used in the clinic or in preclinical toxicological and pharmacological studies must be relatively insensitive to hydrolysis; if it is not, a galenic formulation must guarantee these properties. In the case of budotitane, the use of CremophorEL, a glycerinepolyethyleneglycolericinoleate (BASF), is successful. Budotitane, CremophorEL and propylenglycole are dissolved in water-free ethanol (weight ratio 1 : 9 : 1), the evaporated product is known as a coprecipitate. In order to examine the decomposition of the complex in the watery micellar emulsion, UV-spectra were taken and the levels of intact complex were calculated by component analysis. Fig.6 shows that the major amount (80%) of the micellar emulsion, after a rapid decomposition of 20% of the titanium complex, is stable over many hours in the region from 1 to 2 percent coprecipitate by weight in phys. saline. The initial rapid decomposition must probably be attributed to the destruction of micelles that were defective from the start.

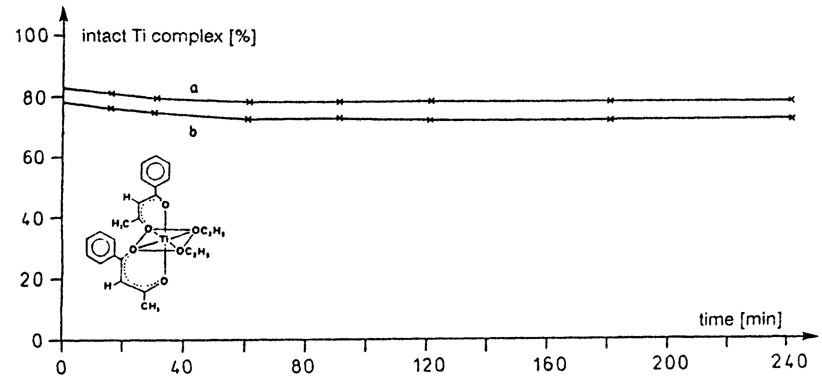

Fig. 6: Time-dependent decomposition of budotitane in an aqueous micellar solution of the coprecipitate. a) 2% b) 1% coprecipitate by weight in isotonic solution.

Finally, the binding behaviour of the before discussed compounds towards DNA is presented in this article. The kinetics of the metal complex-DNA binding have been measured with the help of an ICP-AES (Induced Coupled Plasma-Atom Emission Spectrometer). The binding behaviour of HInd[

$RuCl_4(ind)_2]$ and $HIm[RuCl_4(im)_2]$ (not shown) towards salmon testes DNA gives evidence that similar amounts of metal bind to DNA with similar reaction rates. Interesting but not surprising is the result of these measurements: the titanium complex shows the fastest reaction rate and the best binding, due to its hardness (Fig. 7). It should be noted that both the new tumor-inhibiting complexes budotitane and $HInd[RuCl_4(ind)_2]$ show a high affinity to DNA; especially after one day the measurements show a better binding to DNA than in the case of cisplatin. These results correlate with the presented data of the activity in experimental tumor models and give us hope that the compounds will show good results in the clinical studies.

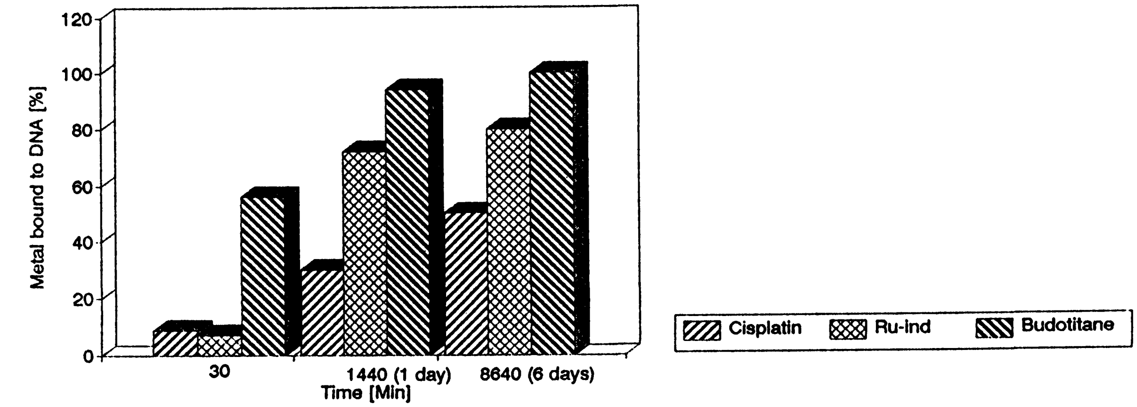

Fig. 7: Binding behaviour of $HInd[RuCl_4(ind)_2]$ and budotitane towards DNA, in comparison with cisplatin. (10 mM NaCl, Salmon Testes DNA)

**Received: June 21, 1993**

